# The effect of diversity on disease reverses from dilution to amplification in a 22-year biodiversity × N × CO_2_ experiment

**DOI:** 10.1038/s41598-024-60725-z

**Published:** 2024-05-13

**Authors:** Alexander T. Strauss, Sarah E. Hobbie, Peter B. Reich, Eric W. Seabloom, Elizabeth T. Borer

**Affiliations:** 1 Department of Ecology, Evolution, and Behavior, University of Minnesota, St. Paul, MN USA; 2 Department of Forest Resources, University of Minnesota, St. Paul, MN USA; 3 Hawkesbury Institute for the Environment, Western Sydney University, Penrith, NSW Australia; 4 Institute for Global Change Biology and School for Environment and Sustainability, University of Michigan, Ann Arbor, MI USA; 5 Present Address: Odum School of Ecology, University of Georgia, Athens, GA USA; 6 Center for the Ecology of Infectious Diseases, University of Georgia, Athens, GA, USA

**Keywords:** Disease, Diversity, Species richness, Dilution effect, Amplification effect, Nitrogen, Carbon dioxide, *Andropogon gerardii*, *Puccinia andropogonis*, Biomass, Community ecology, Ecological epidemiology, Grassland ecology

## Abstract

Plant disease often increases with N, decreases with CO_2_, and increases as biodiversity is lost (i.e., the dilution effect). Additionally, all these factors can indirectly alter disease by changing host biomass and hence density-dependent disease transmission. Yet over long periods of time as communities undergo compositional changes, these biomass-mediated pathways might fade, intensify, or even reverse in direction. Using a field experiment that has manipulated N, CO_2_, and species richness for over 20 years, we compared severity of a specialist rust fungus (*Puccinia andropogonis*) on its grass host (*Andropogon gerardii*) shortly after the experiment began (1999) and twenty years later (2019). Between these two sampling periods, two decades apart, we found that disease severity consistently increased with N and decreased with CO_2_. However, the relationship between diversity and disease reversed from a dilution effect in 1999 (more severe disease in monocultures) to an amplification effect in 2019 (more severe disease in mixtures). The best explanation for this reversal centered on host density (i.e., aboveground biomass), which was initially highest in monoculture, but became highest in mixtures two decades later. Thus, the diversity-disease pattern reversed, but disease consistently increased with host biomass. These results highlight the consistency of N and CO_2_ as drivers of plant disease in the Anthropocene and emphasize the critical role of host biomass—despite potentially variable effects of diversity—for relationships between biodiversity and disease.

## Introduction

Plant communities around the world are simultaneously losing biodiversity and growing under elevated levels of nitrogen (N) and atmospheric carbon dioxide (CO_2_), due to a wide range of human activities including combustion of fossil fuels and use of agricultural fertilizers^1–3^. Critically, biodiversity loss, N, and CO_2_ can all affect the severity of infectious disease in plant communities, such as grasslands^4–9^. However, long-term interactive consequences of these global change drivers on plant disease remain largely unknown.

At the individual plant level, fungal infection severity in grassland taxa often increases with N^10,11^, decreases with CO_2_^12,13^, and decreases with species diversity^4,14^. High concentrations of N in plant tissues can induce host growth at the cost of reduced defense^15^ or promote growth of pathogens^16^ by reducing their stoichiometric mismatches with hosts^17^. Elevated CO_2_ can reduce disease via the same pathway in reverse, diluting nutrients in host tissues with elevated C uptake^4,12,13^, or by reducing stomatal conductance^18^ and exposure to pathogens that enter plants through open stomata^19^. Beyond these abiotic factors, infection risk for individual plants also depends on their community context. Non-hosts can interfere with the transmission of specialist pathogens by blocking spores or attracting vectors away from hosts (i.e., causing ‘dilution effects’)^20–22^. Since these dilution effects can occur in diverse communities but not monocultures, they are often detected as inverse relationships between diversity and disease^14,22–24^. Dilution effects are typically assessed for a single host and pathogen, with “diversity” often quantified as species richness. Thus, a pervasive expectation in disease ecology, supported by meta-analysis^25,26^, is that where diversity declines, disease risk should increase for the hosts that remain.

In addition to these effects that operate on individuals, N, CO_2_, and community context also can shape disease via changes in host biomass. All else equal, higher biomass of plant hosts (or higher density of animal hosts) should accelerate density-dependent disease transmission^27^. Plant biomass often increases with added N or CO_2_ in monocultures but may decrease for taxa in mixtures if they are outcompeted under enriched conditions^5,6,28,29^. Therefore, in addition to changes in host stoichiometry or stomatal conductance, N and CO_2_ could indirectly impact disease via altered host biomass and density-dependent transmission. Critically, effects of species diversity on disease (i.e., dilution effects) also often hinge on host biomass. In addition to physically interfering with transmission, non-hosts often compete with hosts for space or resources, thereby limiting biomass of hosts and the density-dependent spread of their specialist pathogens^22,23,25,30^. An analogous outcome follows logically, although it superficially challenges dilution effect expectations: if hosts reach *higher* biomass in more diverse communities, for example due to facilitation or reduced intraspecific competition, then presence of non-hosts could *amplify* disease^31^. Although biomass-mediated amplification effects like this seem possible, they have not been demonstrated empirically.

Importantly, these abiotic and biotic drivers of disease—those operating on individual hosts and those mediated by host biomass—can become easily conflated. For example, species richness often declines with N, conflating effects of N on disease via enriched tissue chemistry, reduced interference from non-hosts (i.e., weakened dilution effects), and compositional changes in host communities^5,6^. More generally, higher species diversity often correlates with lower biomass of any given host^28^, obscuring whether disease patterns are driven more by host biomass or diversity *per se*^30,32^. Disentangling such effects remains a major challenge. Moreover, while effects of N, CO_2_, and richness on disease are typically assessed as snapshots in time, their impacts may wax or wane as communities undergo long-term compositional change. For example, effects of N and CO_2_ on plant community composition can shift over decades^9,29^, potentially increasing or decreasing host biomass and triggering biomass-mediated changes in disease. Despite this potential for variable effects over time, disease is rarely measured at multiple time points in long-term experiments that manipulate species diversity.

Here, we evaluate how N, CO_2_, and species diversity shaped plant disease severity at the onset of a multi-decadal field experiment and how those patterns changed 20 years later. The experiment (BioCON) has manipulated Biodiversity (i.e., richness of plant communities), CO_2_, and N since it was seeded in 1997^33^. Plots were never reseeded, but planted species were allowed to compete and re-seed themselves, so that their total and relative biomass has changed over time. Disease severity was recorded in the third year (1999), yielding foundational examples of dilution effects and the effects of N and CO_2_ on plant disease^4^. We resampled the experiment two decades later (2019) with nearly identical methods, recording damage severity of a specialist rust fungus (*Puccinia andropogonis*) on its host, big bluestem (*Andropogon gerardii*). Big bluestem is a warm-season (C_4_), perennial grass native to North America, where it can comprise 80% of biomass in tallgrass prairies^34^ and holds bioenergy potential^35^. It typically overyields in mixture^28^ and becomes increasingly dominant over time, both in abandoned agricultural fields^36^ and in the BioCON experiment^29^. The focal pathogen is a heteroecious rust that infects *A. gerardii* as its telial host and bastard toadflax (*Comandra umbellata*) as its aecial host^37,38^. Disease spreads within populations of big bluestem via asexual propagation of urediniospores during the summer.

We divide our analyses into two parts. In Part 1, we ask whether N, CO_2_, and plant diversity affected disease severity similarly in 1999 and 2019. In short, we found that disease increased with N and decreased with CO_2_ in both periods, but that the relationship between diversity and disease reversed from a dilution effect in 1999 to an amplification effect in 2019. In Part 2, we evaluate several explanations for this surprising diversity-disease reversal. We focus on indirect effects of species richness on disease which—if they changed over time—could explain the diversity-disease reversal. Specifically, we evaluate hypotheses related to 1) the plant community (H1A & H1B), 2) microhabitat for the fungal pathogen (H2A & H2B), and 3) soil N supply rates (H3A & H3B).

**H1, Plant Community:** H1A) Disease severity may have increased with higher host biomass^27^, and host biomass may have been higher in monoculture than mixture in 1999 (creating a biomass-mediated dilution effect) but higher in mixture than monoculture in 2019 (creating a biomass-mediated amplification effect). H1B) Disease severity may have decreased with spore-blocking interference by non-hosts, quantified as the probability of interspecific encounter (ENS_PIE_; inverse Simpson’s diversity^22^). Inverse Simpson’s diversity may have increased steeply with richness in 1999 (creating strong dilution effects) but shallowly with richness in 2019 (creating weak or nonexistent dilution effects).

**H2, Microhabitat:** H2A) Disease severity may have decreased with light penetration and H2B) increased with soil moisture, two habitat variables that are important for fungal pathogens^39–42^ and can vary with plant diversity^5,43^.Microhabitat may have been more suitable for the fungus (less light and/or more moisture) in monoculture than mixture in 1999, but more suitable in mixture than monoculture in 2019.

**H3, Soil N Supply:** Disease severity may have increased with soil N supply rates^16,44,45^ (H3A: nitrification; H3B: N mineralization). N supply rates may have been similar in mixture and monoculture in 1999, but 20 years of accumulated biomass of N-fixing legumes may have elevated N supply rates^45,46^—and hence disease—in diverse plots in 2019.

After evaluating each hypothesis, we synthesize them with path analysis. This approach delineates the direct and indirect effects of each treatment on disease in both years. Finally, we examine changes in aboveground biomass of the host in monocultures versus mixture for 25 consecutive years (1998–2022), because it provides the best explanation for the diversity-disease reversal.

## Results

### Part 1: Drivers of disease in 1999 and 2019

**Figure 1:**
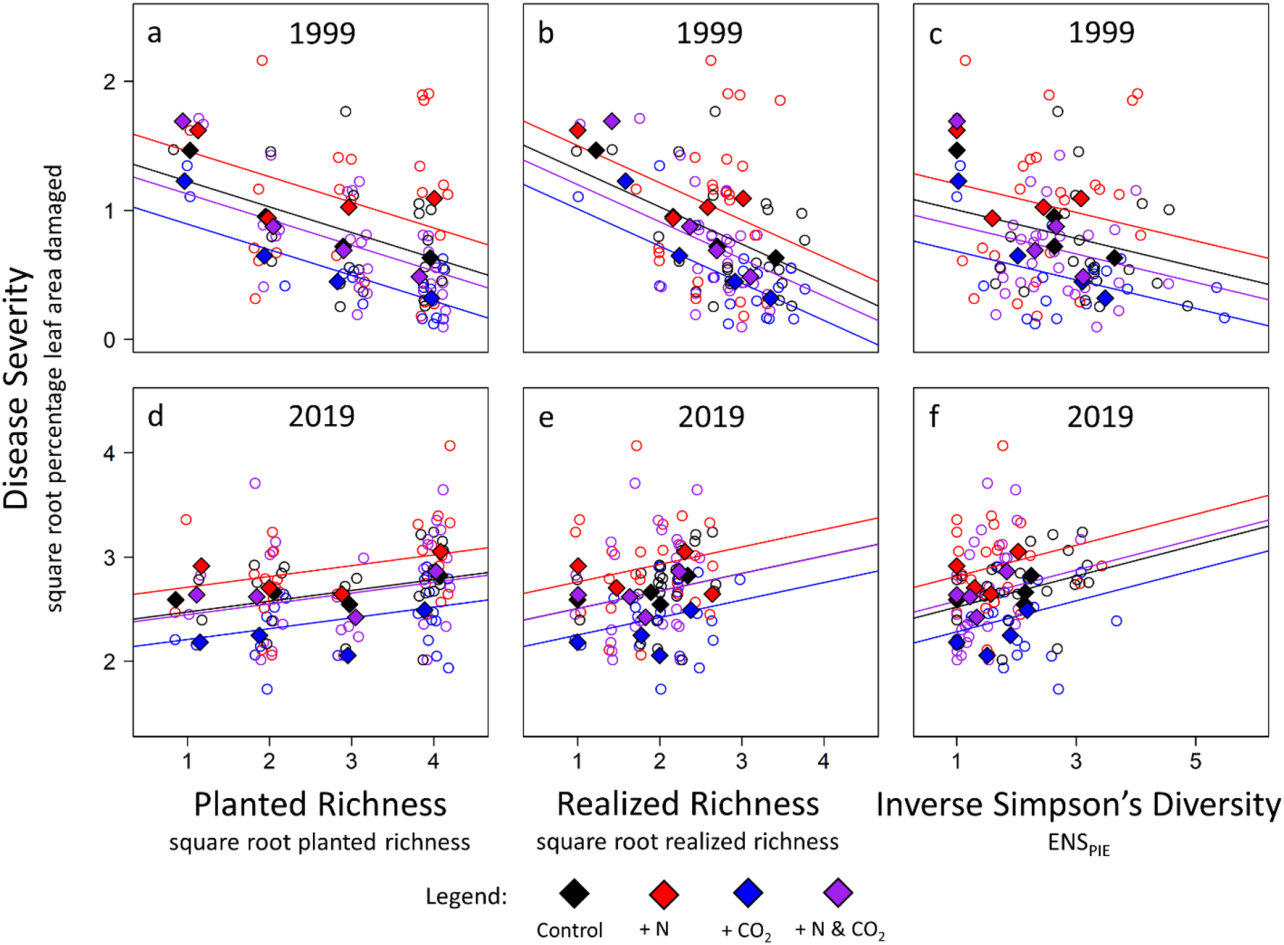
Consistent effects of N and CO_2_ on disease severity but reversal of the diversity-disease relationship in a 20-year field experiment. The host plant (big bluestem, *Andropogon gerardii)* was scored for pathogen damage (rust fungus: *Puccinia andropogonis*) in a long-term field experiment (BioCON) in both 1999 (top) and 2019 (bottom). Treatments, maintained over the twenty-year period, included nitrogen addition (+ N; red), carbon dioxide enrichment (+ CO_2_; blue), addition of both N and CO_2_ (purple), and controls (black). These abiotic treatments were factorially crossed by richness of the plant community (1, 4, 9, or 16 species). Richness treatments were maintained by weeding but not replanted; therefore, realized richness (center) was often a subset of planted richness (left), especially in 2019. Abiotic drivers of disease were consistent: In both 1999 and 2019, disease severity increased with N addition and decreased with CO_2_ enrichment. However, the pattern between diversity and disease reversed. In 1999, disease severity decreased with (**a**) planted richness, (**b**) realized richness, and (**c**) inverse Simpson’s diversity, indicating a ‘dilution effect’. In contrast, (**c**, **d**) disease severity increased with all three metrics of diversity in 2019, indicating an ‘amplification effect’. Richness is jittered for clarity; treatment means shown as solid diamonds; statistics reported in Table 1.

Effects of N and CO_2_ on disease were consistent between sampling periods, but the diversity-disease relationship reversed (Fig. 1; Table 1). Results were qualitatively identical when using planted species richness, realized species richness, or inverse Simpson’s diversity (Table 1; *p* values for realized richness referenced in text). In general, severity of rust disease was greater in 2019 than 1999 (Year effect: *p* = 0.012), perhaps due to background year-to-year variation^37^. With only two years of disease data, we focus instead on the effects of experimental treatments within each period. In both 1999 and 2019, the addition of N increased disease severity (*p* = 0.029). Simultaneously, enrichment of CO_2_ reduced disease severity (*p* = 0.035). Both results agreed with previous studies^4,12^, and effect sizes were strikingly consistent between sampling periods. In contrast, relationships between diversity and disease qualitatively reversed. In 1999, disease severity decreased with plant diversity (Fig. 1a; Appendix Table S3; post-hoc *p* < 0.0001). However, in 2019, disease severity *increased* with diversity (Fig. 1b; post-hoc *p* = 0.049). In other words, the diversity-disease relationship reversed from a dilution effect in 1999 to an amplification effect in 2019, regardless of whether diversity was quantified as planted richness, realized richness, or inverse Simpson’s diversity.

**Table 1:** Effects of N, CO_2_, and plant diversity on disease severity in 1999 and 2019.

Term	DF	Using planted species richness (Fig. 1a,d)			Using realized species richness (Fig. 1b,d)			Using inverse Simpson’s diversity (Fig. 1c,f)		
		Est	SE	*p*-value	Est	SE	*p*-value	Est	SE	*p*-value
Intercept	111	1.44	0.16	< 0.0001	1.61	0.21	< 0.0001	1.09	0.16	< 0.001
Year (2019)	71	0.92	0.20	< 0.0001	0.71	0.28	0.012	1.20	0.22	< 0.001
Diversity	111	− 0.20	0.04	< 0.0001	− 0.29	0.07	0.0001	− 0.10	0.04	0.024
N	111	0.24	0.08	0.0051	0.19	0.09	0.029	0.21	0.09	0.022
CO_2_	4	− 0.33	0.09	0.025	− 0.30	0.10	0.035	− 0.32	0.11	0.044
Year × Diversity	71	0.31	0.05	< 0.0001	0.46	0.11	0.0001	0.280	0.08	< 0.001
Year × N	71	0.00	0.11	0.97	0.07	0.12	0.55	0.11	0.12	0.39
Year × CO_2_	71	0.06	0.11	0.58	0.04	0.11	0.75	0.09	0.12	0.47

In both years, disease severity increased with nitrogen addition (N) and decreased with CO_2_ enrichment (CO_2_). Disease severity decreased with diversity in 1999 but increased with diversity in 2019 (Fig. 1). Model results are qualitatively identical when using planted species richness (left), realized species richness (center), or inverse Simpson’s diversity (right) to quantify “diversity.” Model structure: Disease ~ Year * (Diversity + N + CO2) + (1|Ring/Plot). Year is a factor with two levels (1999 or 2019; default level 1999). Disease severity is square root-transformed to better meet assumptions of normality; species richness is square root-transformed to reduce statistical leverage.

### Part 2: Explaining the diversity-disease reversal

#### Univariate tests

##### H1, Plant Community

**Figure 2:**
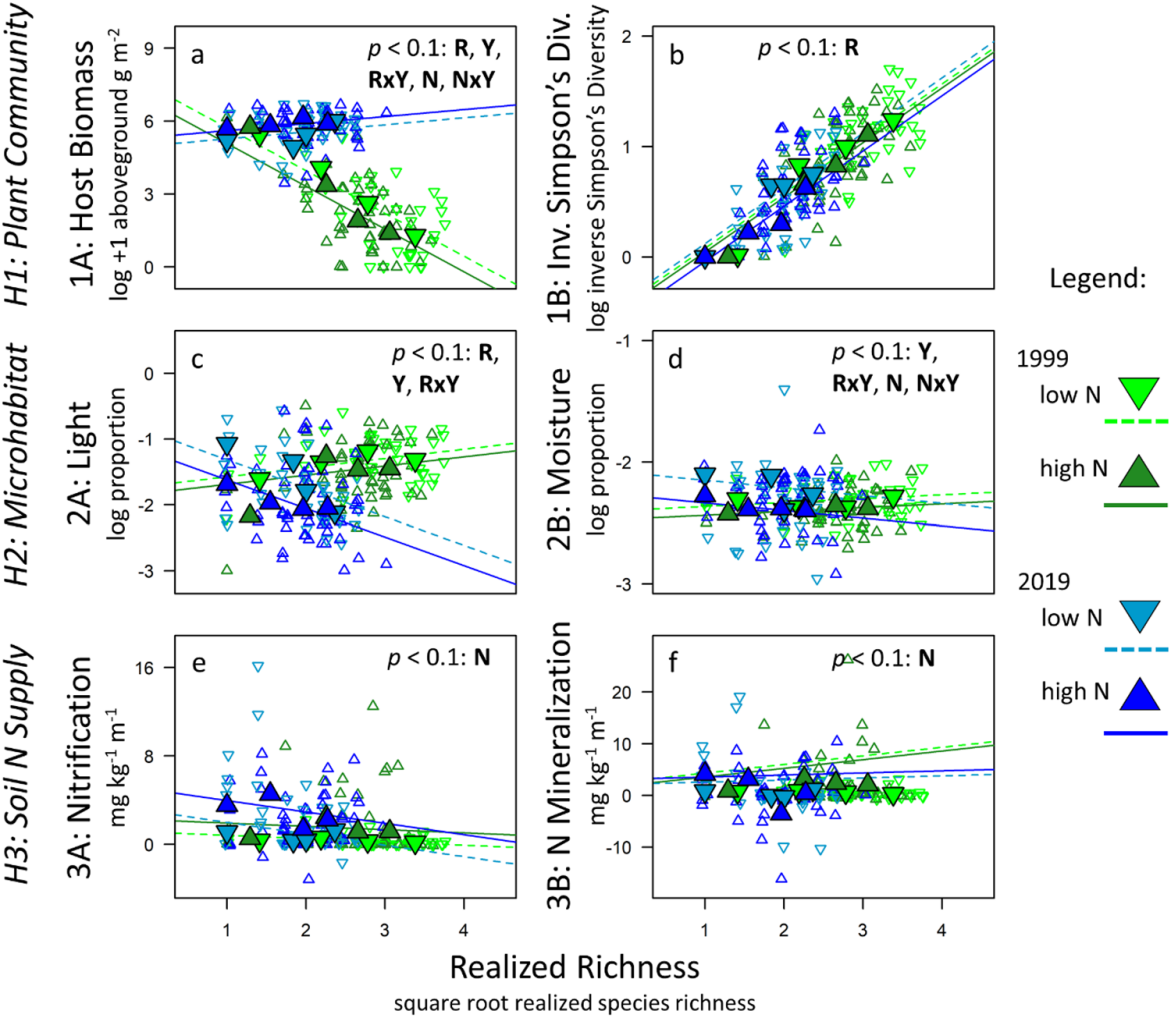
Explaining the diversity-disease reversal: What changed between 1999 and 2019? Reversal of the diversity-disease pattern (Fig. 1) could have resulted from a shift in the underlying relationships between plant diversity and several proximate drivers of disease, including those related to host biomass or the plant community (top row), microhabitat for the fungus (middle row), or soil N supply rates (bottom). Plant Community: We hypothesized that disease severity might increase with aboveground biomass of the host and decrease with inverse Simpson’s diversity. (**a**) Host biomass decreased with richness in 1999 but increased with richness in 2019. Additionally, host biomass decreased with N addition in 1999, but increased with N in 2019. (**b**) Inverse Simpson’s Diversity increased similarly with richness in both years. This response of host biomass—but not inverse Simpson’s diversity—is consistent with the diversity-disease reversal. Microhabitat: We hypothesized that the severity of fungal disease might decrease with light (proportion of light penetrating from canopy to soil) and increase with soil moisture. (**c**) Light decreased with richness in 1999 but increased with richness in 2019. (**d**) Soil moisture slightly increased and decreased with planted richness in 1999 and 2019, respectively, and responded marginally to N. The pattern for light (but not moisture) is consistent with the diversity-disease reversal. Soil N supply: We hypothesized that disease severity might increase with the availability of inorganic N to plant hosts. Although (**e**) nitrification rates and (**f**) net N mineralization rates increased with experimental N addition in both years, neither varied with species richness in either year. Therefore, changes in soil N supply rates cannot explain the diversity-disease reversal. Significant and trending model results (*p* < 0.10) are identified at the top of each panel. Key to abbreviations: R = richness, Y = year, R × Y = Richness × Year interaction, N = Nitrogen, N × Y = Nitrogen × Year interaction.

Aboveground host biomass (H1A) decreased steeply with realized species richness in 1999 (post-hoc *p* < 0.0001) but increased with realized richness in 2019 (post-hoc *p* = 0.032; Fig. 2a; Table S4). This significant year-by-richness interaction (YxR: *p* < 0.0001; Table 2) was consistent with the diversity-disease reversal, since higher host biomass should increase disease severity^27^. Effects of N on host biomass also differed between years (YxN: *p* = 0.0001). N reduced host biomass in 1999 (post-hoc *p* < 0.01) but increased it in 2019 (post-hoc *p* = 0.01; Table S4). Inverse Simpson’s diversity (H1B) was unlikely to contribute to the diversity-disease reversal because it increased with realized species richness with similar steepness in both years (Fig. 2b; Table 2; YxR: p > 0.05). In other words, richness had nearly identical effects on inverse Simpson’s diversity between 1999 and 2019, but qualitatively opposite effects on disease.

**Table 2:** Potential proximate drivers of disease: Summary of statistical tests.

Category of hypothesis	Proximate driver	Predicted effect on disease	*p* value: Year	*p* value: Richness	*p* value: Year × Richness	Other terms with *p* < 0.10	Consistent with diversity-disease reversal?
H1: Plant Community	H1A: Host biomass (Fig. 2a)	↑	0.003	< 0.0001	< 0.0001*	N: *p* = 0.001 N × Y: *p* = 0.001*	**Yes:** Biomass decreases with richness in 1999 but increases with richness in 2019
	H1B: Inv. Simpson’s diversity (Fig. 2b)	↓	ns	< 0.0001	ns		No: Inverse Simpson’s diversity increases similarly with richness in both years
H2: Micro-habitat	H2A: Light (Fig. 2c)	↓	0.012	0.068	< 0.0001*		**Yes:** Light decreases with richness in 2019 but increases with richness in 1999
	H2B: Moisture (Fig. 2d)	↑	0.042	Ns	0.082	N: *p* = 0.097 NxY: *p* = 0.049	No: Soil moisture increases with richness in 1999; decreases with richness in 2019
H3: Soil N Supply	H3A: Nitrification (Fig. 2e)	↑	ns	Ns	ns	N: *p* = 0.030	No: No significant interaction between species richness and year
	H3B: Net N miner-alization (Fig. 2f)	↑	ns	Ns	ns	N: p = 0.045	No: No significant interaction between species richness and year

Summarized statistical tests correspond to relationships shown in Fig. 2. Hypotheses for the diversity-disease reversal are divided into three categories. “Proximate driver” indicates the response variable of a statistical test, with all tests taking the form: Proximate driver ~ Year * (Realized Richness + N + CO_2_) + (1|Ring/Plot). P values indicate effects of Year, Richness, Year x Richness interactions, and any other significant terms (e.g., N = Nitrogen; NxY = Nitrogen x Year interaction). Changes in two of the potential proximate drivers are consistent with the diversity-disease reversal: host biomass and light.

*post-hoc tests determine directionality and significance of each effect in each year (Tables S4 & S5).

##### H2, Microhabitat

Percent light penetration (H2A) increased with realized richness in 1999 (post-hoc *p* = 0.03; Table S5) and decreased with richness in 2019 (post-hoc p < 0.0001; Fig. 2c). This result was also consistent with the diversity-disease reversal because greater light penetration should reduce disease severity^39–42^. Soil moisture (H2B) increased with richness in 1999 (*p* = 0.003) and decreased with richness in 2019 (YxR: *p* = 0.017; Fig. 2d), but we had hypothesized that greater moisture would increase—not decrease—disease.

##### H3, Soil N Supply

Neither soil nitrification rates (H3A; Fig. 2e) nor net N mineralization rates (H3B; Fig. 2f) responded to planted species richness in either year, although both increased significantly with N (Table 2).

#### Synthesis

**Figure 3:**
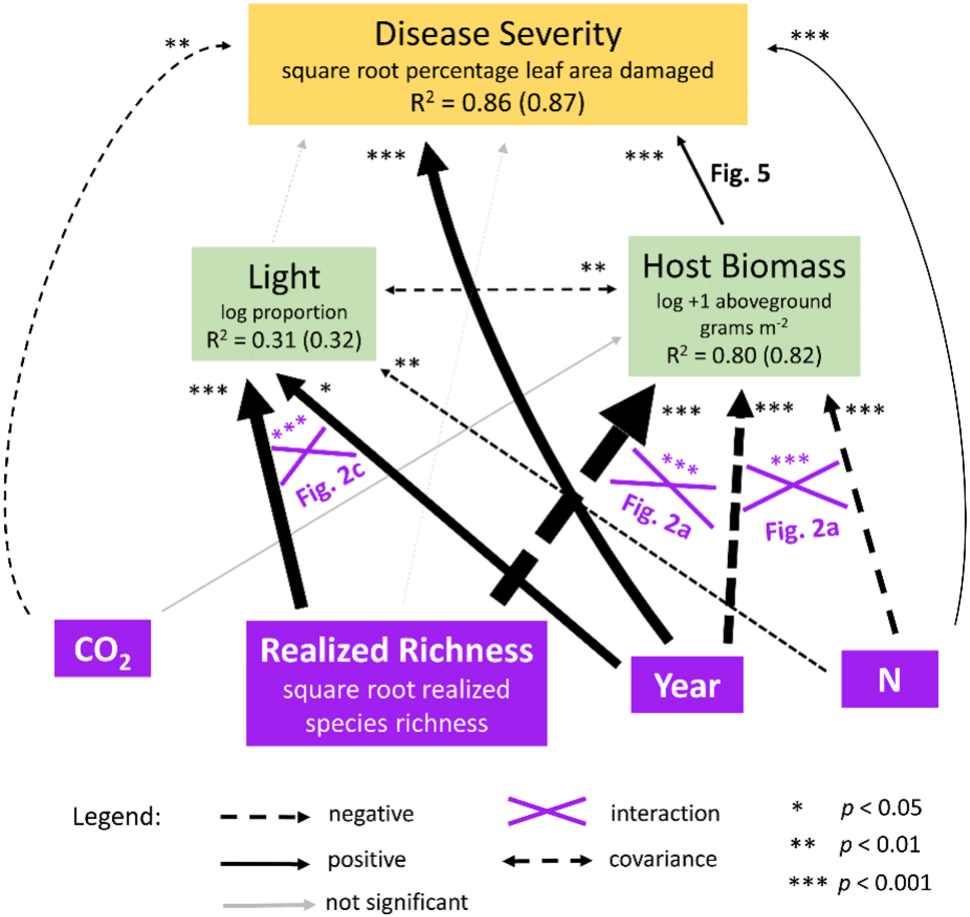
Synthesizing effects of host biomass, light, N, and CO_2_, and richness on disease severity with path analysis. The diversity-disease reversal was best explained by opposite effects of species richness on host biomass between 1999 and 2019. Disease severity consistently decreased with CO_2_ and consistently increased with nitogen and host biomass. In turn, host biomass was shaped by both species richness (negatively in 1999 and positively in 2019) and N (also negatively in 1999 and positively in 2019). Therefore, N shaped disease both directly and indirectly—mediated through changes in host biomass. Light also responded to N, richness, year, and their interaction, but did not shape disease itself. Therefore, patterns of light penetration are unlikely to have contributed substantially to the diversity-disease resersal. On the other hand, after accounting for the reversed relationships between richness and host biomass in 1999 versus 2019, biomass emerged as a consistent driver, increasing disease (Fig. 4). After accounting for this pathway, the direct effect of richness on disease became non-significant. Therefore, the diversity-disease reversal is best explained by a reversal of the effects of species richness on host biomass. Arrow widths are standardized effect sizes; solid arrows indicate positive relationships; dashed arrows indicate negatie relationships; purple crossed lines indicate significant interactions; dotted lines indicate correlations; gray lines show non-significant relationships; asterisks indicate statistical significance; non-significant interactinos not shown. Marginal and conditional R^2^ for endogenous variables are indicated outside and inside parentheses, respectively. Parameters in the appendix (Table S6).

**Figure 4:**
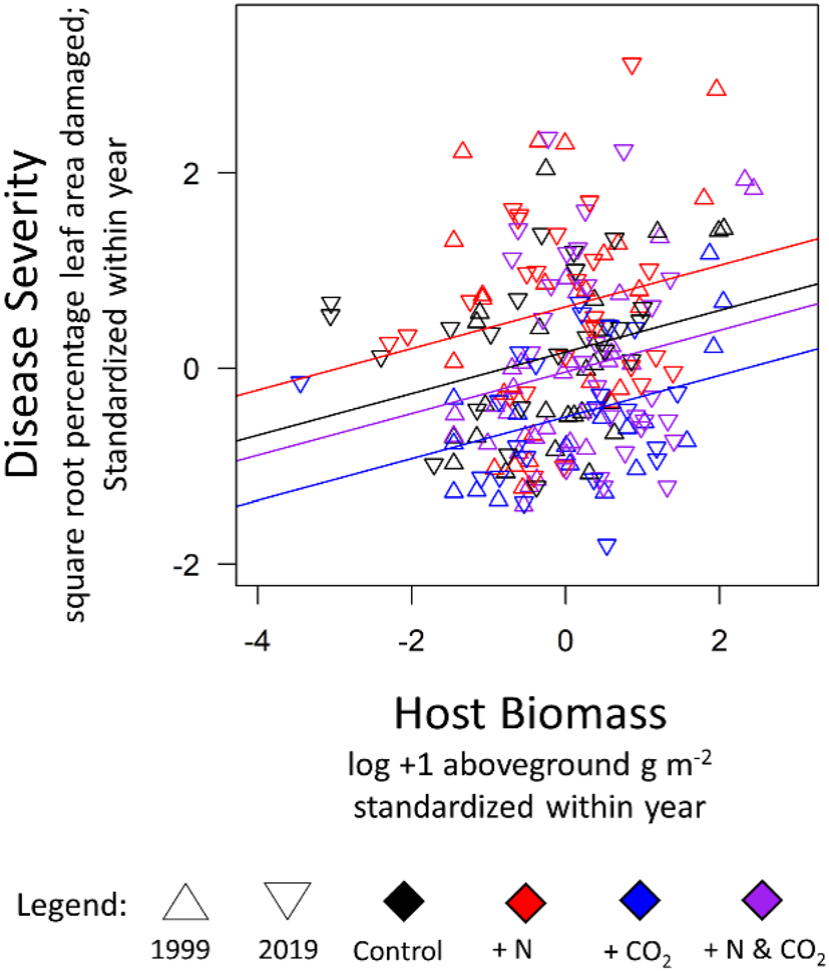
Higher host biomass consistently increases disease severity. Despite the apparent reversal of effects of diversity on disease severity (Fig. 1), higher host biomass is consistently associated with increased disease severity. Both host biomass and disease severity are standardized within year.

Path analysis (Fig. 3) synthesized the two hypotheses that received the strongest support: H1A (host biomass) and H2A (light). The final model fit well (Fisher’s *C* = 0.67; *p* = 0.72 [low *p* value would indicate poor fit]; parameters in Table S6). It explained substantial variation in all endogenous variables, with high conditional and marginal R^2^, respectively, for disease severity (86%; 87%), host biomass (80%; 82%), and light penetration (31%; 32%). Host biomass and light responded to treatments and year-by-treatment interactions as expected from the univariate models (Fig. 2; Table 2). With these foundations in place, the path model revealed which treatments had the strongest effects on disease, and whether effects of N, CO_2_, and richness were mediated by host biomass and/or light. Disease severity was higher in 2019 than 1999 (*p* < 0.0001), decreased with CO_2_ (*p* = 0.006), increased with N (*p* < 0.001), and increased with aboveground host biomass (*p* < 0.0001). In turn, host biomass depended on realized species richness and N, with the direction of these effects differing between years. Species richness and N both reduced host biomass in 1999, exerting indirect biomass-mediated effects that reduced disease severity. In contrast, in 2019, species richness and N both increased host biomass, exerting indirect biomass-mediated effects that increased disease. Disease severity did not respond significantly to light penetration and did not respond directly to realized species richness, after accounting for effects of richness on host biomass. A post-hoc linear model confirmed—in simpler terms—that disease severity increased with host biomass in both years (Fig. 4), suggesting a biomass-mediated dilution effect in 1999 and a biomass-mediated amplification effect in 2019.

#### Long-term trends

**Figure 5:**
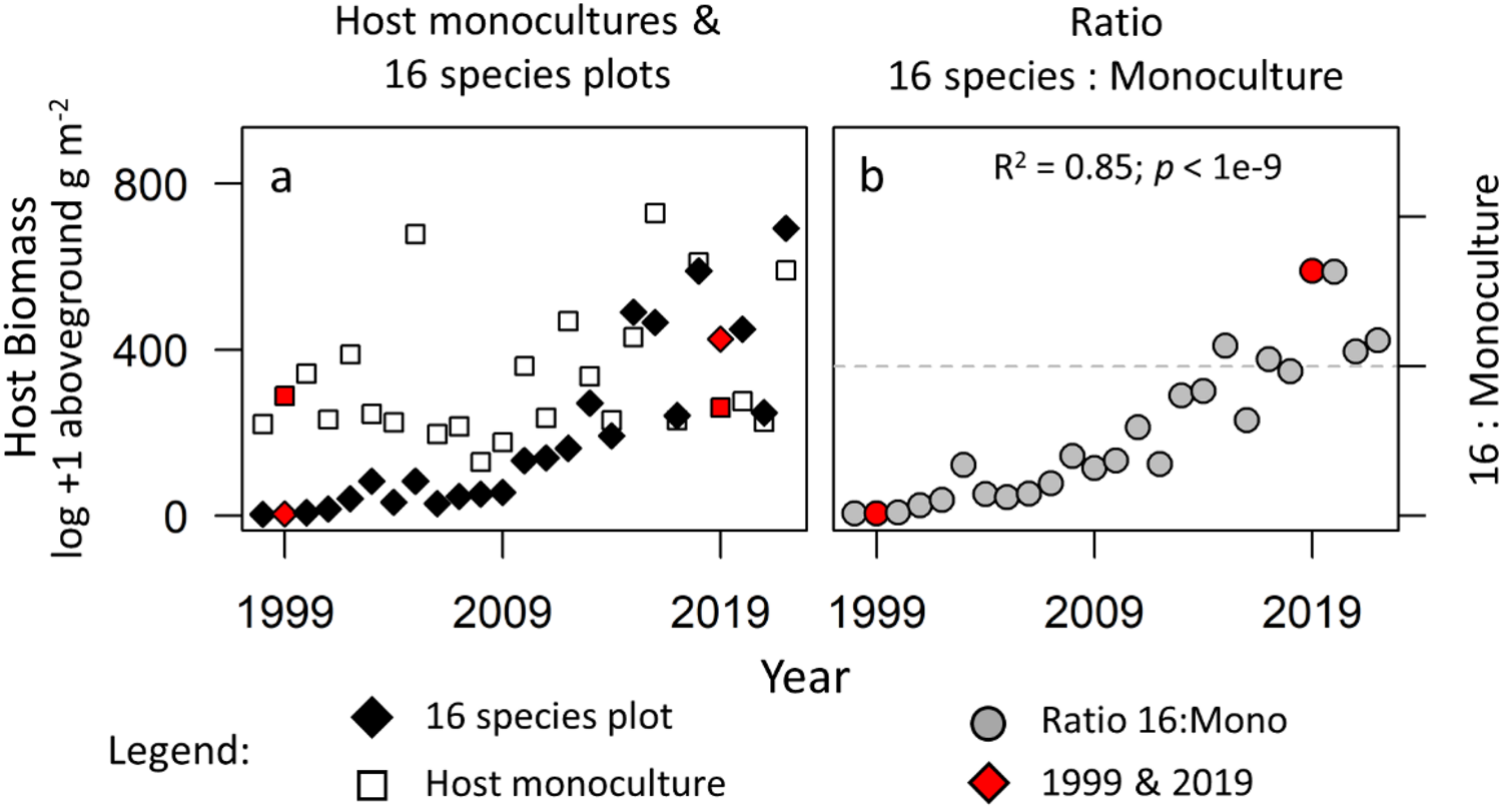
Changes in host biomass as the experiment underwent compositional change over 25 years. Yearly means of aboveground host biomass are shown in (**a**) monocultures (open squares) and diverse 16-species plots (black diamonds), and (**b**) as a ratio between the two (dashed horizontal lines indicating equality), with all means averaged across N and CO_2_ treatments. Red symbols indicate the years in which disease damage was scored. Host biomass was relatively consistent in the monocultures over the 20 years but increased consistently in the 16-species plots over the same period. In 1999, biomass was much higher in the monocultures, but by 2019, biomass was greater in the 16-species plots. Correlation coefficients and *p*-values correspond to simple linear regressions of the depicted relationship.

Aboveground host biomass, measured annually in every plot from 1998 to 2022, increased nearly monotonically in mixtures over the 25-year period (Fig. 5a). Host biomass was initially much greater in monocultures than 16-species plots (Fig. 5b). However, this difference gradually faded over time and eventually reversed in direction, with host biomass in mixture exceeding host biomass in monoculture in six of the eight latest years.

## Discussion

Human endeavors are altering potent abiotic and biotic drivers of plant disease in natural systems^4–6^, but disentangling direct effects of N, CO_2_, and plant diversity from those mediated by changes in host biomass remains challenging. Moreover, any of these effects might shift in strength or even reverse in direction as communities undergo long-term compositional change. Here, we resampled a field experiment that has manipulated nitrogen (N), carbon dioxide (CO_2_), and species richness for over twenty years^29^. We compared drivers of severity of a specialist rust fungus (*Puccinia andropogonis*) on its C4 grass host (*Andropogon gerardii*) in 1999^4^ versus 2019. The net effects of N and CO_2_ were consistent across decades: disease severity increased with elevated N and declined with elevated CO_2_. Effects of N were both direct and indirectly mediated by host biomass. Most notably, the diversity-disease pattern reversed from a dilution effect in 1999 to an amplification effect in 2019. This result was initially surprising, since higher biodiversity typically reduces disease severity, especially for specialist pathogens. We resolved this apparent contradiction by highlighting a consistent positive association between disease and host biomass, as predicted from fundamental epidemiological theory^27^. Thus, diversity itself did not lead to either disease dilution or amplification; rather, patterns between diversity and disease reflected changes in the relative biomass of hosts in mixture versus monoculture.

Our results suggest an underdeveloped mechanism for amplification effects in disease ecology: host augmentation^31^. Dilution effects occur when non-hosts interfere with transmission among hosts in more diverse communities^24^. Non-hosts can inhibit transmission via several prominent mechanisms^22^, most notably ‘host regulation’. In host regulation, non-hosts reduce the biomass or density of hosts, often through competition, and this reduction slows density-dependent transmission of specialist pathogens^23,30^. In turn, amplification effects describe cases when disease severity increases with diversity^22^. Yet mechanisms of amplification of specialist pathogens are much less developed than those for dilution. The most prominent example is ‘vector augmentation’, in which higher diversity increases disease risk by amplifying a population of vectors^47^. Generalist parasites can experience amplification if they can infect multiple species but seek out their preferred host^48^. Here, we identified an important mechanism of amplification for specialist pathogens: host augmentation. In 1999, aboveground biomass of the host was higher in monoculture than mixture, but in 2019 it was higher in mixture than monoculture. In both years, host biomass was positively correlated with disease severity. Thus, host augmentation is the reciprocal of host regulation^31^, resulting in opposite relationships between diversity and disease. We broadly hypothesize that amplification of specialist pathogens along diversity gradients is likely to arise in cases when host biomass increases with biodiversity.

Twenty years of compositional change resulted in the diversity-disease reversal, but reasons for host augmentation in 2019 (i.e., *why* big bluestem achieved higher biomass in mixture than monoculture) remain unclear. Dilution effects are typically assessed as snapshots at a single point in time, but important dynamics may only arise after hosts and non-hosts interact for multiple generations. For example, if hosts are stronger interspecific competitors and they replace non-hosts over multiple generations, non-hosts become less likely to interfere with transmission and dilute disease as they become rarer^23,30^. Although we presented disease data from only two timepoints, the twenty-five years of biomass data allowed us to track the steady increase in dominance of the host in diverse plots. Specifically, the host reached higher biomass in monocultures for the first 17 years of the experiment but achieved higher biomass in mixture for six of the next eight years. We explored whether this ‘host augmentation’ effect could be explained by the percent cover of legumes in mixture^45^ and regenerated soil fertility^46^ but did not find any suggestive patterns. The host does typically benefit from reduced intraspecific competition in mixture (i.e., ‘overyields’)^28^ and becomes increasingly dominant with successional age^49^. Accumulation of host-specific soil pathogens in monocultures (but not yet mixture) may have contributed as well^50,51^. Interestingly, this switch in relative biomass between monoculture and mixture means that the same species that caused biomass-mediated dilution effects in 1999 caused biomass-mediated amplification effects in 2019. This observation again reiterates the critical importance—and simplicity—of host biomass for mediating diversity-disease relationships. If the relationship between diversity and host biomass changes over time, so too can the relationship between biodiversity and disease.

Net effects of N consistently increased disease, CO_2_ reduced it, and the combined effects of N and CO_2_ together offset one another almost exactly. One interpretation is that N enriched host tissue chemistry and fueled pathogen growth by reducing the stoichiometric mismatch between hosts and pathogens^17^, while CO_2_ diluted plant tissue N and intensified this mismatch^12^. Independent measurements of big bluestem tissue chemistry in the experiment confirmed that percent N in leaves did increase with N fertilization but did not respond to CO_2_ enrichment^52^. Thus, host stoichiometry might explain some of the effects of N on disease (i.e., those that are not mediated by biomass) but is less likely to explain the effects of CO_2_. An alternative explanation for the CO_2_ effects involves stomata. Hosts that respond to elevated CO_2_ by reducing stomatal conductance can benefit from less disease, since many fungal pathogens enter leaves through open stomata^19^. Big bluestem reduced its stomatal conductance by 23% under elevated CO_2_, which is modest relative to other species’ responses^18^, but may have nevertheless reduced its exposure to spores of this rust fungus. Future paired measurements of stomatal conductance, tissue chemistry, and disease severity might yield more mechanistic insights into these effects of N and CO_2_ on disease that operate on individual hosts.

Although the net effect of N was similar between 1999 and 2019, the indirect effects of N mediated by host biomass reversed in direction. Specifically, N reduced host biomass in 1999—indirectly limiting disease severity via density dependent transmission—but increased host biomass in 2019, indirectly elevating disease. This switch likely occurred because other plants benefited from additional N when the host was rare in mixture in 1999. In contrast, the host benefited from N when it had become more dominant in 2019. The reversal of these biomass-mediated effects also suggests that effects of N on disease that operated on individual hosts (e.g., via tissue chemistry) must have been stronger in 1999 and weaker in 2019, since net effects of N were similar. This result is consistent with the resource-ratio hypothesis of succession, which posits that N becomes relatively less limiting as succession proceeds^49^. While the experiment did not undergo succession per se, compositional shifts in the diverse plots are broadly consistent with successional dynamics in neighboring old fields^29,36^. Thus, N fertilization might have increased individual-level disease more strongly at the onset of the experiment—when N may have been more limiting for both the host and pathogen—than after 20 years of succession-like compositional change. Fertilization experiments and disease measurements along successional gradients would be need to test this hypothesis directly.

The consistent effects of N and CO_2_ that we observed may have been different if we had examined other host and pathogen taxa. Here, severity of *Puccinia* fungus on a C4 grass host increased with N and decreased with CO_2_. Meta-analysis shows that fertilization frequently increases the severity of fungal foliar diseases, especially those caused by *Puccinia* and *Pythium*^11^. In a globally distributed field experiment, N consistently increased fungal disease severity in grasses but reduced disease severity in forbs^10^. In the 1999 survey in BioCON, N increased disease severity in C4 grasses, but not C3 grasses, legumes, or forbs^4^. Thus, N increases disease more consistently in grasses than in other functional groups. Stoichiometry might explain this pattern: Since grasses typically have higher C:N than other functional groups^53^, perhaps they impose greater stoichiometric mismatches for their pathogens, and perhaps those pathogens respond more positively to N, as has been shown in other hosts^17^. Effects of CO_2_ on disease seem less related to host functional group. In 1999 in BioCON, elevated CO_2_ *increased* disease severity of C3 grasses in monoculture^4^. In other years, elevated CO_2_ *reduced* foliar fungal disease severity on the forb *Solidago rigida*^7^, the legume *Lespedeza capitata*^8^, and the C4 focal host of this study, *Andropogon gerardii*. Future analyses should investigate whether known interspecific differences in stomatal responses^18^ explain any of this variation. Any patterns that link traits of hosts, e.g., related to functional group or stomatal conductance, could become valuable tools for predicting responses of plant diseases to the changing conditions of the Anthropocene.

Future investigations could expand our results in several important directions. First, direct manipulation of host biomass in similarly sized plots would enable a more direct test of the effects of biomass on disease severity. Second, collection of disease damage in additional years could reveal the importance of climatic variables for this fungus, which is known to vary among years^37^. More years of disease data could also enable tests of whether severe disease in one year regulates host biomass in the next, and consistency of the relationship between host biomass and disease. Third, measurements of disease severity on all hosts in the experiment^4^—not only *A. gerardii*—could enable us to scale up our results to more diverse natural communities. We would expect that effects of N and CO_2_ might vary by functional group or other host traits (e.g., stomatal conductance), and that any hosts that achieved lower biomass in mixture than monoculture should generally experience dilution effects for their specialist pathogens. Similarly, we would expect any other taxa that reach higher biomass in mixture to experience disease amplification. Importantly, patterns of community structure and biomass are likely to change over time for all species—as we demonstrated here for big bluestem.

By resampling a field experiment after 20 years, we found that two important abiotic drivers of global change—N and CO_2_—caused consistent and opposite net effects on the severity of plant disease. In contrast, effects of a key biotic driver of disease—plant diversity—reversed in direction from a dilution effect in 1999 to an amplification effect in 2019. This diversity-disease reversal was consistent with a directional shift in plant community composition, with hosts initially reaching higher biomass in monoculture than mixture but eventually achieving higher biomass in mixture than monoculture. Nitrogen also had increasingly positive effects on host biomass over time. Ultimately, we explained the diversity-disease reversal by documenting the consistently positive associations among host biomass, density-dependent transmission, and disease. As plant communities continue to lose biodiversity and receive elevated N and CO_2_, it becomes increasingly important to recognize that some consequences of these global changes—especially those mediated by host biomass—may only manifest after decades. In the case of dilution and amplification effects, inconsistent relationships between diversity and disease may instead reflect changes in the dominance of hosts within diverse communities over time.

## Methods

### Part 1: Drivers of disease in 1999 and 2019

The BioCON experiment at Cedar Creek Ecosystem Reserve (https://biocon.umn.edu/) manipulates plant biodiversity as planted species richness (1, 4, 9, or 16 species per plot), CO_2_ (ambient or + 180 parts per million), and N (ambient or + 4 g N m^−2^ year^−1^ as slow-release anhydrous ammonium nitrate) in a full factorial design^4,33^. Soil at the site is sandy (> 90% sand) and N-poor. CO_2_ enrichment is maintained using Free Air CO_2_ Enrichment (FACE) technology, with 4 m^2^ experimental plots distributed into six spatial ‘rings’ (3 ambient; 3 enriched) for a split-plot design. In 1997, for each of the four combinations of CO_2_ and N, 32 plots were seeded with a single species (monocultures: two plots per plant species at each combination of CO_2_ and N; eight total monoculture plots per plant species), 15 plots were seeded with four species, 15 plots were seeded with 9 species, and 12 plots were seeded with 16 species. All 16 species were native or naturalized to perennial prairies, and included four C_4_ grasses (*Andropogon gerardii* [big bluestem; the focal host of this study], *Bouteloua gracilis*, *Schizachyrium scoparium*, and *Sorghastrum nutans*), four C_3_ grasses (*Agropyron repens*, *Bromus inermis*, *Koeleria cristata*, and *Poa pratensis*), four legumes (*Amorpha canescens*, *Lespedeza capitata*, *Lupinus perennis*, and *Petalostemum villosum*), and four non-leguminous forbs (*Achillea millefolium*, *Anemone cylindrica*, *Asclepias tuberosa*, and *Solidago rigida*). Since the year of planting, each plot was weeded 2–4 times every growing season to remove species that were not originally planted. Species that went locally extinct in a plot were not reintroduced. The present analysis includes all plots where *A. gerardii* was seeded and observed and did not receive other experimental treatments (Table S1). The 1999 analysis included 94 plots and excluding 28 plots where the host was planted but not observed. The 2019 analysis included 100 plots and excluding 22 plots that received warming or rain removal treatments beginning in 2012.

By 1988, the second year of the experiment, most plant species had been colonized by specialist foliar fungal pathogens^4^. In this region, the host big bluestem is conspicuously and consistently infected by a macrocyclic, heteroecious rust fungus, *Puccinia andropogonis*^4,54^*.* Rust fungi are wind-dispersed obligate biotrophic pathogens, typically heteroecious and host-specific on their grass (telial) host^55^. The aecial host of *P. andropogonis* is bastard toadflax (*Comandra umbellata*)^38,54^. Disease severity on big bluestem increases with proximity to bastard toadflax due to dispersal of aeciospores, although this effect fades beyond 40 m^37^. Infected big bluestem then produces urediniospores, which reinfect big bluestem and propagate rapidly. Since bastard toadflax is not planted in BioCON, we assume that all big bluestem in the experiment were equally exposed to aeciospores, and that differences in disease severity we observed were driven by propagation of urediniospores within the local big bluestem population. Although effects of infection by this pathogen on fitness of big bluestem have not been directly quantified, fungicide experiments at Cedar Creek Ecosystem Reserve reveal that exclusion of foliar fungi in monocultures^51^ and communities dominated by big bluestem results in higher plant biomass^56^ and elevated photosynthetic rates^57^.

In both 1999 and 2019, severity of rust disease was scored on big bluestem when it reached its peak biomass in early August. Disease severity was assessed as the percent of leaf area visibly damaged by fungus^10,23^, using standardized digitized reference cards^58^. In 1999, up to 50 leaves were randomly assessed per plot, depending on availability of host leaves^23^. In 2019, we randomly selected 10 host tillers per plot and assessed percent damage on one newer leaf (fully emerged) and one older leaf (not yet senesced) per tiller (20 leaves inspected per plot). We doubled the replication for monoculture plots (20 tillers; 40 leaves) since these plots were less well replicated in the experiment (8 total plots). Disease data were collected within two weeks, in a randomized order of plots within rings, to avoid systematic biases related to any human-mediated rust dispersal. In both years, disease damage data were averaged at the plot level.

We tested whether effects of N, CO_2_, and diversity on disease severity were consistent between periods with linear mixed effects models, using the nlme package^59^ in R version 3.5.2^60^. Disease damage and species richness were square root transformed. Fixed effects included year (1999 or 2019), each treatment (N, CO_2_, and diversity as a continuous factor), and all two-way interactions between year and each treatment. Random effects included ring and plot within ring (intercepts only) to reflect the split-plot design, and since most plots were sampled both years. Separate models considered three different metrics of “diversity”: planted species richness (i.e., the number of species seeded into each plot in 1997), realized species richness (i.e., the number of species observed in the cover subplot each year), and inverse Simpson’s diversity (calculated from the cover subplot). AIC was used to confirm suitability of models where disease varied linearly (rather than quadratically) with square-root-transformed richness or inverse Simpson’s diversity (Table S2). Post-hoc tests (omitting fixed effects of year and random effects of plot) confirmed the direction of the diversity-disease relationship in each year (Table S3).

### Part 2: Explaining the diversity-disease reversal

Since disease differed across levels of richness that were manipulated in the experiment, richness was the ultimate driver of disease. However, its impacts could manifest through several different proximate pathways, related to (1) the plant community, (2) microhabitat for the fungus, and (3) soil N supply. For each hypothesis, we ask whether the potential proximate driver of disease responded differently to diversity between years. Then, we use path analysis to synthesize the supported hypotheses and delineate direct and indirect effects of treatments on disease. Finally, using 25 years of continuously collected data, we investigate how host aboveground biomass—the most likely driver of the diversity-disease reversal—varied as the experiment underwent compositional change. Although the diversity-disease reversal was consistent when quantifying diversity as planted richness, realized richness, or inverse Simpson’s diversity, all subsequent analyses use realized richness since it was explicitly manipulated in the experiment and provides a more accurate representation of diversity than planted richness.

#### Hypothesis 1, Plant Community: host biomass (H1A) and inverse Simpson’s diversity (H1B)

Higher host biomass should accelerate density-dependent disease transmission^23,27^. Additionally, transmission interference by non-hosts should be more likely in communities where hosts are more likely to encounter non-hosts^14,20,22^. We used inverse Simpson’s Diversity (also known as the probability of interspecific encounter; ENS_PIE_)^61^ to test this hypothesis, since this metric of diversity is designed specifically to reflect the probability of interspecific encounter. Host aboveground biomass and inverse Simpson’s diversity were assessed in August of every year in every plot. Inverse Simpson’s diversity was calculated from percent cover in a 0.5 m × 1 m subplot in the center of each plot. Aboveground biomass was harvested from a 1 m × 10 cm strip, dried to a constant mass, and weighed. Nonplanted species (i.e., weeds) were excluded since their regular removal made them unlikely to impact disease dynamics. Biomass was sorted to species in monocultures, 9-species plots, and 16-species plots. For 4-species plots, we interpolated host biomass using a linear regression that predicted host biomass from total biomass and percent cover of the host. This approach provided a strong fit, explaining 90% of the variation in host biomass in plots where biomass had been sorted to species.

#### Hypothesis 2, Microhabitat: light (H2A) and moisture (H2B)

Nonhost species also can impact disease dynamics by altering the suitability of microhabitat for pathogens. For example, inclusion of glutinous rice in agricultural fields altered the microclimate in a way that reduced severity of fungal disease on glutinous rice^43^. Similarly, in a grassland field experiment, plant diversity affected fungal disease severity most strongly via changes in humidity^5^. Fungal pathogens tend to grow best under warm, moist, shaded conditions^39–42^. In general, species richness could impact these habitat variables in dense communities that block light and inhibit evaporation. Light penetration and soil moisture were measured in every plot repeatedly during the growing season in each year. We used light and soil moisture data collected in July, since they were most relevant (collected shortly before disease damage was assessed). Light was measured using Decagon Accupar light meters in the same subplot used to quantify percent cover. Measurements were recorded above the canopy and above the previous year’s litter (averaged among three locations), with percent light penetration calculated as their ratio. Soil moisture was measured in 2-inch PVC pipes inserted in the northeast corner of every plot. Measurements were taken using Time Domain Reflectometry (TDR) from 0 to 20 cm from 1998 to 2001 and from 0 to 17 cm from 2002 to 2019^62^.

#### Hypothesis 3, Soil N Supply

Because fertilization with abiotic N consistently increased disease severity (Fig. 1), we hypothesized that species richness impacted disease indirectly via a ‘hidden treatment’ (sensu Huston ^44^) related to soil N supply rates. In a neighboring experiment, net N mineralization rates were higher in monocultures of legumes and forbs than grasses^63^. Although the focal host here is a grass that cannot fix N, legumes are present in most of the more diverse plots. Moreover, over the first 13 years of BioCON, species richness had increasingly positive impacts on soil N mineralization rates over time^45^. Nitrification and N mineralization were recorded in every plot in every year using the semi-open core method. In late June or early July, four soil cores (20 cm deep; 2 cm diameter) were removed, while two PVC tubes (20 cm long; 2 cm diameter) were placed in each plot and capped with rubber stoppers. The four soil cores were composited, sieved (2 mm), and extracted with 1 M KCl to determine initial concentrations of ammonium- and nitrate–N (Alpkem autoanalyzer). After 30 days, the two PVC tubes were removed, composited, and their soils processed in the same way. Net N mineralization rates were calculated by subtracting total inorganic N (ammonium and nitrate) in the four initial soil cores from those in the field-incubated PVC tubes. Nitrification rates were calculated similarly but used only nitrate data.

### Statistical analyses

We used linear mixed models to ask how each of these potential proximate drivers of disease responded to realized species richness, and whether these relationships differed between 1999 and 2019. Separate models used, as response variables, host aboveground biomass (H1A), inverse Simpson’s diversity (H1B), light penetration (H2A), soil moisture (H2B), nitrification (H3A), and net N mineralization (H3B). Each model included N, CO_2_, realized richness, year, and interactions between treatment and year as fixed effects, with plot and ring included as nested random effects. The year-by-treatment interactions tested whether realized richness (or any other treatments) had different effects on the response variables between years. Response variables were log transformed where appropriate. Two proximate drivers changed in ways consistent with the diversity-disease reversal: host biomass and light. Post-hoc models confirmed the directionality of these effects in each year (Tables S4 & S5).

We employed path analysis using the package piecewiseSEM^64^ to synthesize the supported univariate hypotheses. We initially constructed a path model that included direct effects of N, CO_2_, realized species richness, year, and both potential proximate drivers (host biomass and light) on disease, interactions between each treatment and year on host biomass, and an interaction between richness and year on light. This model structure allowed us to delineate direct effects of treatments from those mediated by changes in host biomass and identify the most parsimonious explanation for the diversity-disease reversal. Direct effects were specified as generalized linear models, with separate models for each endogenous variable (disease severity, host biomass, and light). Variables were log transformed and rescaled where appropriate. Nested random effects were included for ring and plot. We used tests of directed separation^64^ to identify other pathways whose inclusion would improve model fit. Through this process, we added an effect of N on light and a correlation between light and host biomass because both pathways were biologically sensible. We then evaluated the final model with Fisher’s *C* statistic and extracted standardized parameter estimates and R^2^ values for each endogenous variable (both marginal and conditional).

Since the path model emphasized the importance of host biomass, we fit a simpler linear model to test whether host biomass had consistent effects on disease in both 1999 and 2019. Both host biomass and disease severity were standardized (i.e., converted to z-scores) within year. Host biomass, N, and CO_2_ were fixed effects, with plot and ring as nested random effects.

Finally, we used simple linear regression to ask how mean host biomass in 16-species plots relative to monocultures changed over time the 25-year duration of the experiment.

### Supplementary Information

Supplementary Information.

## Data Availability

All data and code will be made publicly available on Dryad upon acceptance. Data may also be requested via personal communication with the corresponding author, ATS.
