# Supplementary material for: The effect of diversity on disease reverses from dilution to amplification in a 22-year biodiversity × N × CO2 experiment
Source: Sci Rep. 2024 May 13;14:10938. doi: 10.1038/s41598-024-60725-z (PMC11091214; doi:10.1038/s41598-024-60725-z)
Supplement: Supplementary file 1 — Supplementary Information. [file 41598_2024_60725_MOESM1_ESM.docx]

**The effect of diversity on disease reverses from dilution to amplification in a 22-year biodiversity x N x CO_2_ experiment**

Alexander T. Strauss*^1,2^ (atstrauss@uga.edu), Sarah E. Hobbie^1^ (shobbie@umn.edu), Peter B. Reich^3,4^ (preich@umn.edu), Eric W. Seabloom^1^ (seabloom@umn.edu), and Elizabeth T. Borer^1^ (borer@umn.edu)

^1^Department of Ecology, Evolution, and Behavior, University of Minnesota, St. Paul, MN, USA

^2^present address: Odum School of Ecology, University of Georgia, Athens, GA, USA

^3^Department of Forest Resources, University of Minnesota, St. Paul, MN, USA

^4^Hawkesbury Institute for the Environment, Western Sydney University, Penrith, New South Wales, Australia

**APPENDIX**

In this appendix, we provide additional details of plots used in the analysis (Table S1). We also use AIC to evaluate whether diversity has linear or non-linear effects on root-transformed richness and Simpson’s diversity (Table S2), provide results for the post-hoc models that determine the direction and significance of the relationship between diversity and disease severity in each year separately (Table S3), post-hoc models that determine the direction and significance of effects of N and realized richness on host biomass in each year separately (Table S4), and post-hoc models that determine the direction and significance of effects of realized richness on light penetration in each year separately (Table S5). Finally, we provide all parameter estimates from the final path model (Table S6).

**Table S1.** Design of the BioCON experiment and plots included in the analyses. For each combination of N, CO_2_, and planted species richness, numbers in each cell indicate the total number of plots in the overall experiment (“plots”), the number of plots used in analysis of the 1999 data (“1999”), and the number of plots used in analysis of the 2019 data (“2019”). Reasons why some plots were not used are explained in the footnotes.

| N & CO_2_ Treatment | Planted species richness | | | |
| --- | --- | --- | --- | --- |
|  | 1 | 4 | 9 | 16 |
| Ambient N & Ambient CO_2_ | *plots:* 32*  *1999:* 2*  *2019:* 2* | *plots:* 15  *1999:* 3^†^  *2019:* 7^†^ | *plots:* 15^†^  *1999:* 7^†^  *2019:* 3^†,‡^ | *plots:* 12  *1999:* 11^¶^  *2019:* 12 |
| Ambient N & Enriched CO_2_ | *plots:* 32  *1999:* 2  *2019:* 2 | *plots:* 15  *1999:* 2  *2019:* 6 | *plots:* 15  *1999:* 4  *2019:* 1^‡^ | *plots:* 12  *1999:* 9^¶^  *2019:* 12 |
| Enriched N & Ambient CO_2_ | *plots:* 32  *1999:* 1  *2019:* 2 | *plots:* 15  *1999:* 6  *2019:* 11 | *plots:* 15  *1999:* 6  *2019:* 1^‡^ | *plots:* 12  *1999:* 11^¶^  *2019:* 12 |
| Enriched N & Enriched CO_2_ | *plots:* 32  *1999:* 2  *2019:* 2 | *plots:* 15  *1999:* 5  *2019:* 9 | *plots:* 15  *1999:* 12  *2019:* 6^‡^ | *plots:* 12  *1999:* 11^¶^  *2019:* 12 |

*Only monocultures of the focal host, *Andropogon gerardii*, were used; monocultures of other plant species were not used in the present analyses.

†Plots planted with 4 or 9 species were only included if the focal host was both planted in the plot and if aboveground biomass was observed in the year in which disease damage was scored. Aboveground biomass of the host was not observed in many of the plots where it was sown in 1999.

‡Several plots planted with 9 species received warming or rain removal treatment beginning in 2012, and were therefore removed from the 2019 analysis

¶ The focal host was planted in all 16-species plots, but aboveground biomass was not observed in all plots in 1999.

**Table S2.** Evaluation of nonlinear effects of diversity on disease severity using AIC. Lower values of AIC indicate stronger model performance (bolded). Only fixed effects are shown. Models with linear effects of diversity on disease outperform models with quadratic effects of diversity on disease in all three cases, whether diversity is quantified as planted species richness, realized species richness, or inverse Simpson’s diversity.

| Fixed Effects (Linear Mixed Models) | # Fixed Effects | Log  Likelihood | AIC |
| --- | --- | --- | --- |
| **Year x (N + CO_2_ + Planted Richness)** | **8** | **-108.83** | **239.66** |
| Year x (N + CO_2_ + Planted Richness) + (Year x Planted Richness^2^) | 10 | -107.89 | 241.77 |
| **Year x (N + CO_2_ + Realized Richness)** | **8** | **-111.90** | **245.81** |
| Year x (N + CO_2_ + Realized Richness) + (Year x Realized Richness^2^) | 10 | -115.17 | 256.34 |
| **Year x (N + CO_2_ + Inverse Simpson’s Diversity)** | **8** | **-117.06** | **256.12** |
| Year x (N + CO_2_ + Inverse Simpson’s) + (Year x Inverse Simpson’s^2^) | 10 | -118.55 | 263.10 |

**Table S3.** Effects of diversity on disease severity, separately for 1999 and 2019. These post-hoc tests are necessary because the significant year-by-diversity interactions reported in the main text (Table 1) merely indicate that the relationship between diversity and disease differed between years; not necessarily that it reversed from dilution (significant negative coefficient) to amplification (significant positive coefficient).

| **1999** | *Using Planted Richness* | | | | *Using Realized Richness* | | | | *Using Inverse Simpson’s* | | | |  |  |  |  |  |
| --- | --- | --- | --- | --- | --- | --- | --- | --- | --- | --- | --- | --- | --- | --- | --- | --- | --- |
| Term | Est. | SE | DF | *p*-value | Est. | SE | DF | *p*-value | Est. | SE | DF | *p*-value |  |  |  |  |  |
| Intercept | 1.42 | 0.18 | 86 | <0.0001 | 1.60 | 0.23 | 86 | <0.0001 | 1.11 | 0.18 | 86 | <0.0001 |  |  |  |  |  |
| Diversity | -0.19 | 0.04 | 86 | <0.0001 | -0.29 | 0.07 | 86 | 0.0001 | -0.11 | 0.04 | 86 | 0.016 |  |  |  |  |  |
| N | 0.23 | 0.08 | 86 | 0.006 | 0.19 | 0.09 | 86 | 0.031 | 0.20 | 0.09 | 86 | 0.030 |  |  |  |  |  |
| CO_2_ | -0.33 | 0.15 | 4 | 0.087 | -0.30 | 0.16 | 4 | 0.12 | -0.32 | 0.17 | 4 | 0.13 |  |  |  |  |  |
|  |  |  |  |  |  |  |  |  |  |  |  |  |  |  |  |  |  |
| **2019** | *Using Planted Richness* | | | | *Using Realized Richness* | | | | *Using Inverse Simpson’s* | | | |  |  |  |  |  |
| Term | Est. | SE | DF | *p*-value | Est. | SE | DF | *p*-value | Est. | SE | DF | *p*-value |  |  |  |  |  |
| Intercept | 2.37 | 0.14 | 92 | <0.0001 | 2.34 | 0.20 | 92 | <0.0001 | 2.37 | 0.16 | 92 | <0.0001 |  |  |  |  |  |
| Diversity | 0.10 | 0.04 | 92 | 0.005 | 0.17 | 0.08 | 92 | 0.049 | 0.15 | 0.07 | 92 | 0.026 |  |  |  |  |  |
| N | 0.24 | 0.08 | 92 | 0.003 | 0.25 | 0.08 | 92 | 0.002 | 0.30 | 0.08 | 92 | <0.001 |  |  |  |  |  |
| CO_2_ | -0.26 | 0.11 | 4 | 0.075 | -0.26 | 0.12 | 4 | 0.092 | -0.24 | 0.11 | 4 | 0.090 |  |  |  |  |  |

**Table S4.** Effects of realized species richness, N, and CO_2_ on host biomass, separately for 1999 and 2019.

| **1999** |  | | | |  |  |  |  |  |
| --- | --- | --- | --- | --- | --- | --- | --- | --- | --- |
| Term | Estimate | SE | DF | *p*-value |  |  |  |  |  |
| Intercept | 7.23 | 0.64 | 86 | <0.0001 |  |  |  |  |  |
| Richness | -1.76 | 0.19 | 86 | <0.0001 |  |  |  |  |  |
| N | -0.66 | 0.24 | 86 | 0.0073 |  |  |  |  |  |
| CO_2_ | 0.39 | 0.45 | 4 | 0.44 |  |  |  |  |  |
|  |  |  |  |  |  |  |  |  |  |
| **2019** |  | | | |  |  |  |  |  |
| Term | Estimate | SE | DF | *p*-value |  |  |  |  |  |
| Intercept | 4.86 | 0.31 | 92 | <0.0001 |  |  |  |  |  |
| Richness | 0.28 | 0.13 | 92 | 0.032 |  |  |  |  |  |
| N | 0.32 | 0.12 | 92 | 0.010 |  |  |  |  |  |
| CO_2_ | 0.32 | 0.21 | 4 | 0.19 |  |  |  |  |  |

**Table S5.** Effects of realized species richness, N, and CO_2_ on light penetration, separately for 1999 and 2019.

| **1999** |  | | | |  |  |  |  |  |
| --- | --- | --- | --- | --- | --- | --- | --- | --- | --- |
| Term | Estimate | SE | DF | *p*-value |  |  |  |  |  |
| Intercept | -1.70 | 0.22 | 86 | <0.0001 |  |  |  |  |  |
| Richness | 0.15 | 0.07 | 86 | 0.029 |  |  |  |  |  |
| N | -0.13 | 0.08 | 86 | 0.13 |  |  |  |  |  |
| CO_2_ | -0.06 | 0.15 | 4 | 0.70 |  |  |  |  |  |
|  |  |  |  |  |  |  |  |  |  |
| **2019** |  | | | |  |  |  |  |  |
| Term | Estimate | SE | DF | *p*-value |  |  |  |  |  |
| Intercept | -0.82 | 0.26 | 92 | 0.0019 |  |  |  |  |  |
| Richness | 0.46 | 0.10 | 92 | <0.0001 |  |  |  |  |  |
| N | -0.31 | 0.09 | 92 | 0.0012 |  |  |  |  |  |
| CO_2_ | -0.00 | 0.22 | 4 | 1.00 |  |  |  |  |  |

**Table S6.** Parameters of the final path model, shown graphically in Fig. 3. Test statistics: Fisher’s C = 0.667 with *p* = 0.72 on 2 degrees of freedom. Richness is realized richness.

| Response | Predictor | Estimate | SE | DF | *p* value | Standardized estimate |
| --- | --- | --- | --- | --- | --- | --- |
| **Disease severity** | Light | -0.08 | 0.06 | 72 | 0.21 | -0.04 |
| R^2^ = 0.86 (0.87) | CO_2_ | -0.32 | 0.06 | 4 | 0.0058 | -0.15 |
|  | Host biomass | 1.15 | 0.28 | 72 | 0.0001 | 0.23 |
|  | N | 0.22 | 0.06 | 111 | 0.0006 | 0.11 |
|  | Year | 1.46 | 0.10 | 72 | 0.0001 | 0.69 |
|  | Richness | -0.00 | 0.06 | 72 | 0.94 | -0.00 |
| **Host biomass** | Year | -0.25 | 0.06 | 71 | 0.0002 | -0.59 |
| R^2^ = 0.80 (0.82) | Richness | -0.38 | 0.04 | 71 | 0.0001 | -1.23 |
|  | N | -0.17 | 0.04 | 111 | 0.0002 | -0.39 |
|  | CO_2_ | 0.05 | 0.05 | 4 | 0.38 | 0.12 |
|  | Year x Richness | 0.21 | 0.02 | 71 | 0.0001 | 0.97 |
|  | Year x N | 0.10 | 0.03 | 71 | 0.0003 | 0.40 |
|  | Year x CO_2_ | -0.01 | 0.03 | 71 | 0.72 | -0.04 |
| **Light** | Richness | 0.67 | 0.18 | 73 | 0.0004 | 0.83 |
| R^2^ = 0.31 (0.32) | Year | 0.65 | 0.29 | 73 | 0.026 | 0.60 |
|  | N | -0.21 | 0.07 | 111 | 0.0018 | -0.20 |
|  | Year x Richness | -0.54 | 0.12 | 73 | 0.0001 | -0.99 |
| Correlation: Host biomass ~~ Light | | -0.17 | na | 194 | 0.0077 | -0.17 |
